# Optimal price subsidies for appropriate malaria testing and treatment behaviour

**DOI:** 10.1186/s12936-016-1582-1

**Published:** 2016-11-04

**Authors:** Kristian Schultz Hansen, Tine Hjernø Lesner, Lars Peter Østerdal

**Affiliations:** 1 Department of Global Health and Development, London School of Hygiene and Tropical Medicine, 15-17 Tavistock Place, London, WC1H 9SH UK; 2 Department of Public Health, University of Copenhagen, Øster Farimagsgade 5, 1014 Copenhagen, Denmark; 3 Department of Business and Economics, and Centre of Health Economics Research (COHERE), University of Southern Denmark, Campusvej 55, 5230 Odense M, Denmark; 4 Department of Economics, Copenhagen Business School, Porcelænshaven 16A, 2000 Frederiksberg, Denmark

**Keywords:** Malaria, Drugs, Diagnostics, Subsidies, Treatment-seeking, Private sector

## Abstract

**Background:**

Malaria continues to be a serious public health problem particularly in Africa. Many people infected with malaria do not access effective treatment due to high price. At the same time many individuals receiving malaria drugs do not suffer from malaria because of the common practice of presumptive diagnosis. A global subsidy on artemisinin-based combination therapy (ACT) has recently been suggested to increase access to the most effective malaria treatment.

**Methods:**

Following the recommendation by World Health Organization that parasitological testing should be performed before treatment and ACT prescribed to confirmed cases only, it is investigated in this paper if a subsidy on malaria rapid diagnostic tests (RDTs) should be incorporated. A model is developed consisting of a representative individual with fever suspected to be malaria, seeking care at a specialized drug shop where RDTs, ACT medicines, and cheap, less effective anti-malarials are sold. Assuming that the individual has certain beliefs of the accuracy of the RDT and the probability that the fever is malaria, the model predicts the diagnosis-treatment behaviour of the individual. Subsidies on RDTs and ACT are introduced to incentivize appropriate behaviour: choose an RDT before treatment and purchase ACT only if the test is positive.

**Results:**

Solving the model numerically suggests that a combined subsidy on both RDT and ACT is cost minimizing and improves diagnosis-treatment behaviour of individuals. For certain beliefs, such as low trust in RDT accuracy and strong belief that a fever is malaria, subsidization is not sufficient to incentivize appropriate behaviour.

**Conclusions:**

A combined subsidy on both RDT and ACT rather than a single subsidy is likely required to improve diagnosis-treatment behaviour among individuals seeking care for malaria in the private sector.

**Electronic supplementary material:**

The online version of this article (doi:10.1186/s12936-016-1582-1) contains supplementary material, which is available to authorized users.

## Background

Malaria continues to be a major cause of mortality and morbidity with 214 million cases and 438,000 deaths worldwide in 2014. The majority of all deaths (90%) occurred in Africa and with 74% of these in children below 5 years [1]. Malaria deaths are largely avoidable, as a broad range of effective and cost-effective tools for prevention and cure of malaria exists. The cost of prevention per disability-adjusted life year averted ranges between US$27–143 [2]. The current manufacturer price of artemisinin-based combinations, the most effective anti-malarials on the market, is about US$2 for an adult course and US$0.5 for a treatment course for a child under five while the less effective chloroquine costs US$0.05–0.15 [3]. Huge investments by governments and international donors over the last 10 years have contributed to the decrease in malaria mortality rates by 25% globally and 33% in Africa [4].

One major obstacle to bringing the disease burden further down is the widespread problem of inappropriate treatment of malaria. Many people infected with malaria do not receive an effective anti-malarial (the access problem) while a large proportion of people receiving treatment for malaria does not suffer from malaria (the targeting problem).

Public health sectors in many countries offer free malaria treatment services, but access is impaired by frequent stock-outs of drugs, short opening hours, long travel distances and prescribed anti-malarials are not always artemisinin-based combinations [5–8]. Therefore, it is common behaviour in many African countries to seek malaria treatment in the private sector especially at small, specialized drug shops and general stores [9, 10]. The price of artemisinin-based combination therapy (ACT) may be 10–15 times higher than other anti-malarials and many customers instead buy cheaper but much less effective monotherapies, sub-therapeutic doses or no anti-malarials at all [5, 11–14]. Common anti-malarial monotherapies include chloroquine, sulfadoxine-pyrimethamine (SP) and quinine [5].

Targeting effective drugs to those who are truly suffering from malaria is hampered by the widespread use in many countries of presumptive diagnosis rather than more accurate parasitological tests leading to overdiagnosis of malaria and underdiagnosis of other diseases [15, 16]. The proportion of parasitological testing among patients treated for malaria was estimated to be 47% in the public sector in the African Region in 2011 [8] with a considerably lower testing rate in the private sector—possibly one-third of the public sector and even less frequently in drug shops [17]. Studies across different countries and settings have documented that between 30 and 80% of people treated with an anti-malarial do not have malaria parasites in their blood [18–25].

With an objective of improving access to high quality ACT medicines, both in the public and private sectors, a global subsidy paid directly to accredited ACT manufacturers was proposed in the early 2000s and subsequently operationalized under the name of ‘the Affordable Medicines Facility-malaria (AMFm)’ and hosted by the Global Fund [26, 27]. Pilot tests in several malaria endemic countries found that such a subsidy achieved considerable success in terms of increasing availability of ACT, hugely reducing the retail price differences between ACT and older, less effective monotherapies in the private sector and increasing the sales volume of ACT medicines [28–31].

Subsidizing ACT medicines may increase access but it may also lead to increased treatment of patients not suffering from malaria. The World Health Organization now recommends that all suspected malaria cases should be confirmed with a parasitological test before treatment and that positive cases should be treated with an ACT [32]. Accurate rapid diagnostic tests (RDTs) for malaria have recently been developed which are easy to use with immediate result, require only limited training of providers and could feasibly be sold and performed in drug shops and other private sector outlets [8, 33, 34].

The AMFm idea of a global subsidy on ACT has recently been abandoned to consider alternative, possibly more cost-effective interventions including increased focus on introducing RDTs. In the meantime, individual malaria stricken countries may still apply for funds to finance ACT medicines and even RDTs from the Global Fund [35]. Cohen et al. [22] conducted a randomized controlled trial in rural Kenya to assess the impact of changing both RDT and ACT prices through the use of subsidies. They found that ACT use increased 59% in presence of a subsidy of 90%—but only 56% of those buying ACT test positive for malaria. However, they also found that targeting increased to 81% when the subsidy for ACT was slightly reduced (from 90 to 80%) and the freed resources directed to an RDT subsidy of 85% instead. This increased the testing rate more than 50% and had no significantly negative effect on ACT uptake.

In this paper the characteristics of an optimal subsidy policy will be investigated when a health planner has the objective that suspected malaria patients should be diagnosed and treated according to WHO guidelines. The focus is on the private sector, in particular private drug retailers. These are an extremely important source of anti-malarial treatment and the problem of inappropriate treatment of malaria is common in terms of frequent sale of less effective drugs (non-artemisinins) and parasitological testing being the exception rather than the rule. An analytical framework is developed based on expected utility theory where a representative individual with suspected malaria has to make a choice at a drug shop regarding purchasing an RDT and a type of anti-malarial. The framework also contains a health planner who can influence the prices of RDTs and ACT at drug shops using subsidies. Optimal subsidy levels for RDTs and ACT are explored within this framework and supplemented by numerical simulations to investigate the influence of key factors such as the prior belief of the individual that the fever is due to malaria as well as his/her trust in the accuracy of RDTs. The results from this framework suggest that exclusively subsidizing ACT, as proposed by the AMFm approach, is in general not sufficient for incentivizing the individual to behave as desired by the health planner. A price reduction on RDTs is necessary as well and the optimal use of subsidy funds is a combined subsidy on RDT and ACT. The present paper complements the paper by Cohen et al. [22] by explicitly modelling both the subsidy choices of a public health planner and the household decision making by households. This framework enables a search for an ‘optimal’ combination of RDT and ACT subsidy levels.

## Methods

### Model of individual behaviour in malaria treatment-seeking in the private sector

A simple decision model is developed where a representative febrile individual can choose among different strategies involving choice of drugs and whether to take a parasitological test before treatment. The focus is here on malaria treatment and testing strategies and there are two possible health states: The individual either has malaria or not malaria. $V_{m}$ is the utility of having malaria and $V_{nm}$ is the utility of not having malaria with $V_{nm} > V_{m}$. The utilities $V_{m}$ and $V_{nm}$ may be thought of as expressing monetary values so that $V_{nm} - V_{m}$ is the willingness to pay to avoid malaria. The individual does not know for certain whether the fever is malaria or not but holds a belief *p* (a subjective probability) that the fever is malaria. This belief is affected by the result of an RDT. Define $p_{p}$ as the belief that a fever is malaria having observed that the RDT result is positive, whereas $p_{n}$ is the belief that a fever is malaria having observed that the RDT result is negative. It is assumed that $p_{n} < p < p_{p}$, so that a positive RDT result will increase the individual’s belief that the fever is malaria while a negative RDT result will decrease the belief that the fever is caused by malaria. If the individual has complete confidence in the accuracy of the test, i.e. believes that there are no false positive or false negative test results, then $p_{p}$ will be equal to 1 and $p_{n}$ will be equal to 0. Let us call $p^{*}$ the individual’s belief that the test result will be positive. From $p$, $p_{p}$ and $p_{n}$ the following can be defined $p = p^{*} p_{p} + \left( {1 - p^{*} } \right)p_{n}$, and therefore

$$p^{*} = (p - p_{n} )/(p_{p} - p_{n} ).$$

The belief $p^{*}$ may not necessarily be equal to $p$ if for instance the individual is concerned that the RDT will occasionally miss positive malaria cases (false negatives) in which case $p^{*}$ will be lower than $p$. Similarly, the individual holds beliefs that two available types of drugs, monotherapy and ACT, will cure malaria, $E_{MT}$ and $E_{ACT}$ where $E_{ACT} > E_{MT}$. The retail prices of the drugs are denoted $C_{MT}$ and $C_{ACT}$, where $C_{ACT} > C_{MT}$, and with the price of the test denoted $C_{RDT}$. Values of beliefs $p$, $p_{n}$, $p_{p}$, $E_{MT}$ and $E_{ACT}$ fall between 0 and 1 while prices of drugs and RDT are positive.

**Fig. 1:**
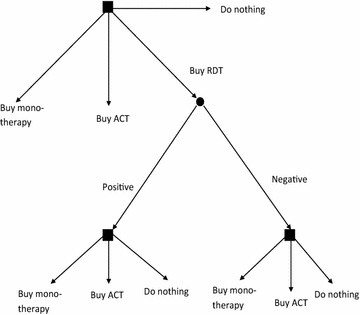
Diagnosis-treatment strategies

One possible strategy for the individual is to do nothing about the fever if it is believed to be self-resolving, a strategy that will be denoted $S_{NO}$, another is that the individual seeks treatment at a drug shop or another private health provider if he believes the fever to be caused by malaria. While it is a possibility that the fever is caused by a serious non-malarial disease, the focus is here on whether it is malaria or not and it is assumed that the individual will seek care at formal providers in case a fever is expected to be a serious non-malarial disease. In the drug shop, the individual faces the following options: (a) buy cheap, less effective antimalarial monotherapy such as chloroquine or SP, strategy $S_{MT}$, (b) buy more effective but also more expensive ACT, strategy $S_{ACT}$ or (c) buy a rapid diagnostic test (RDT) and let the subsequent decision of buying an ACT medicine, monotherapy or no drugs depend on the result of the test. The decision to purchase an RDT will lead to nine possible strategies. One example of a strategy is that the individual purchases a cheap anti-malarial monotherapy if the RDT is positive and does not buy any anti-malarials if the RDT is negative, strategy $S_{(MT,NO)}^{RDT}$. The possible strategies of the individual are represented graphically in Fig. 1.

All possible strategies involve risky outcomes and it is assumed that the individual chooses the strategy with the highest expected utility *U*. The expected utility of buying no drugs and without having a test is:

$$U(S_{NO} ) = pV_{m} + (1 - p)V_{nm}$$

The expected utility of not purchasing a test or drugs is therefore the belief that the fever is malaria times the utility of having malaria plus the belief that the fever is not malaria times the utility of being free of malaria. The expected utility of buying a cheap anti-malarial monotherapy without having a test is:

$$U(S_{MT} ) = pE_{MT} V_{nm} + p(1 - E_{MT} )V_{m} + (1 - p)V_{nm} - C_{MT}$$

The expected utility is the probability of being cured for malaria after taking monotherapy times the utility of being malaria free (first term) plus the probability of monotherapy not working times the utility of having malaria (second term) plus the belief of the fever not being malaria times the utility of being malaria free (third term). In addition, the retail price of anti-malarial monotherapy must be subtracted. Likewise the expected utility of buying an ACT medicine without having a test is:

$$U(S_{ACT} ) = pE_{ACT} V_{nm} + p(1 - E_{ACT} )V_{m} + (1 - p)V_{nm} - C_{ACT}$$

with a similar interpretation as above.

The utility of a strategy of buying first an RDT followed by the purchase of a course of ACT if the test is positive and not purchase any drugs if the test is negative is:

$$\begin{aligned} U\left( {S_{{(ACT,NO)}}^{{RDT}} } \right) &= p^{*} \left[ p_{p} E_{{ACT}} V_{{nm}} + p_{p} \left( {1 - E_{{ACT}} } \right)V_{m} \right. \\ &\; \qquad \left. + \left( {1 - p_{p} } \right)V_{{nm}} - C_{{ACT}} \right] \\& \quad + \left( {1 - p^{*} } \right)\left[ {\left( {1 - p_{n} } \right)V_{{nm}} + p_{n} V_{m} } \right] - C_{{RDT}} \\ \end{aligned}$$

The first component of the expected utility consists of the belief that the RDT will be positive, $p^{*}$, times the utility of taking a course of ACT and with a belief that the fever is malaria adjusted upwards from $p$ to $p_{p}$. The second component is the belief that the RDT will be negative, $(1 - p^{*} )$, times the utility of not taking any anti-malarials and with a belief that the fever is malaria adjusted downwards from $p$ to $p_{n}$. Finally, the third component is the RDT price, $C_{RDT}$, which must be subtracted. The expected utility function for the remaining eight RDT-strategies arising from the decision tree in Fig. 1 can be written in a similar fashion (Additional file 1).

Some of the possible strategies are not rational. Consider a strategy consisting of first purchasing an RDT associated with a decision to purchase an ACT medicine irrespective of the test result ($S_{(ACT,ACT)}^{RDT}$). It would make more sense to save the money for purchasing an RDT and instead go directly to acquiring an ACT medicine: The strategy $S_{(ACT,ACT)}^{RDT}$ is dominated by the strategy $S_{ACT}$. It also seems irrational to choose a strategy of buying the most effective and expensive drug only when the test is negative like the strategy $S_{(MT,ACT)}^{RDT}$. It can be shown formally that six such strategies are suboptimal (see Additional file 2 for details). Consequently, a rational individual will choose from the remaining six strategies: $S_{ACT}$, $S_{MT}$, $S_{NO}$, $S_{(ACT,NO)}^{RDT}$, $S_{(MT,NO)}^{RDT}$ and $S_{(ACT,MT)}^{RDT}$.

### The objective of the health planner

A health policy planner is now introduced who wants the current malaria treatment guidelines as recommended by WHO to be followed: All suspected malaria cases must be diagnosed with a parasitological test before treatment and patients with confirmed malaria should be treated with an ACT while patients with a negative test should not receive an anti-malarial [32]. An individual visiting a drug shop does not necessarily behave according to the guidelines. For instance, if the expected utility for the individual of strategy $S_{ACT}$ is higher than the expected utility of strategy $S_{(ACT,NO)}^{RDT}$ then the individual will purchase an ACT directly rather than following the strategy advised by the health planner. However, the health planner could potentially reverse the ranking of these two strategies by changing the relative prices of ACT and RDT through subsidies. This will be the case if a combination of subsidies can be found such that the utility of strategy $S_{(ACT,NO)}^{RDT}$ is higher than the utility of strategy $S_{ACT}$, when the prices are reduced due to the subsidies. Similar conditions are needed to ensure that the utility of strategy $S_{(ACT,NO)}^{RDT}$ is higher than the remaining four non-eliminated strategies. There are therefore five conditions which are presented in Additional file 3 as inequalities (1)–(5).

There may be more than one combination of ACT and RDT subsidy levels ensuring that the individual prefers strategy $S_{(ACT,NO)}^{RDT}$ to all other strategies. The health planner therefore has as an objective that the total subsidy cost should be minimized subject to the constraint that the treatment guidelines are followed. In general, total subsidy cost for the health planner of a combination of subsidy levels is:

6 $${\text{Total subsidy cost}} = \beta^{ACT} *\tilde{p} + \beta^{RDT}$$

where $\beta^{ACT}$ is the subsidy cost per ACT course, $\beta^{RDT}$ is the subsidy cost per RDT and $\tilde{p}$ is the probability that an RDT will be positive which depends on the malaria parasite prevalence among individuals visiting drug shops and the accuracy of the RDT. Positive RDT results will include both true and false positives and $\tilde{p}$ can be written as

$$\tilde{p} = \bar{p}*ss^{RDT} + \left( {1 - \bar{p}} \right)*(1 - sp^{RDT} )$$

where $\bar{p}$ is the malaria parasite prevalence among febrile individuals visiting drug shops while $ss^{RDT}$ and $sp^{RDT}$ are the sensitivity (probability of a positive test for an infected person) and specificity (probability of a negative test result for an uninfected person) respectively of the RDT.

The decision problem of the health planner consists of minimizing total subsidy cost (6) over subsidy levels for ACT and RDT subject to inequalities (1)–(5) listed in Additional file 3 being simultaneously obeyed.

### Searching for optimal RDT and ACT subsidies: individual beliefs and numerical simulations

There is no general solution to this optimization problem as it will depend on specific values of prices and parameters. Therefore, an approach is followed where a series of numerical examples will give indications on what combinations of subsidies on ACT and RDT incentivize appropriate behaviour and have the lowest total subsidy cost for the health planner. Two sets of numerical assumptions are applied related to (1) prices and RDT accuracy and (2) beliefs of the individual with fever.

(1) The retail prices, drug effectiveness and RDT accuracy listed in Table 1 are intended to represent ‘the average’ or ‘a common’ situation in sub-Saharan Africa. It is assumed in the numerical examples that subsidies will directly change retail prices corresponding to an assumption that the subsidy is perfectly passed on to individuals visiting private sector providers. For instance, if a subsidy is 75%, then the individual will pay only 25% of the pre-subsidy price. This approach to subsidization in the analysis may therefore be interpreted as a subsidy on the retail prices facing the individual in contrast to the AMFm approach where the subsidy is given to ACT medicine manufacturers at the top of the supply chain [28].

**Table 1:** Retail prices excluding subsidies and parameter values used in numerical simulations

Description	Parameter	Best estimate	Lower bound	Upper bound	Source
Retail price of monotherapy^#,&^	$$C_{MT}^{{}}$$	US$0.3	US$0.1	US$1.6	[3, 44]
Retail price of ACT^#^	$$C_{ACT}^{{}}$$	US$3.5	US$1.0	US$10.0	[3, 17]
Retail price of RDT	$$C_{RDT}^{{}}$$	US$1.8	US$0.5	US$2.93	[3, 17]
Effectiveness of monotherapy^&^	$$E_{MT}$$	50%	20%	70%	[45, 46]
Effectiveness of ACT	$$E_{ACT}$$	95%	90%	99%	[47]
RDT sensitivity	$$ss^{RDT}$$	95%	86%	99%	[48, 49]
RDT specificity	$$sp^{RDT}$$	95%	75%	99.8%	[49]

^#^ This price guide reports manufacturer prices and in order to arrive at estimates of retail prices a mark-up of 100% is assumed as found in a market survey [50]

^&^ Non-artemisinin monotherapy

In the model above, monetary (US$) retail prices are converted into a price comparable to the utility model using a linear transformation where the monetary prices are divided with the individual’s willingness to pay (WTP) for avoiding malaria illness. Unfortunately, an empirical estimate of such a WTP does not exist. Instead a contingent valuation survey from Uganda is relied on which found an average WTP for an adult course of ACT of US$2.05 among drug shop customers who were asked their valuation of a course of ACT after having purchased an RDT that turned out positive [36]. Because this is a WTP for a specific drug to cure malaria and not as such a WTP to avoid malaria in the first place, the estimate of US$2.05 is considered as a lower bound and a WTP of US$3.00 is used as the best guess.

(2) An individual may hold different beliefs with respect to the fever being malaria ($p$) and change this belief after RDT testing ($p_{n}$ and $p_{p}$). Large differences between $p$ on the one hand and $p_{n}$ and $p_{p}$ on the other indicate high trust in the RDT result. There is evidence that people have strong beliefs in a positive test result but the belief in a negative test result typically varies and can be quite low [37, 38]. Methods have recently been developed that may be used to elicit empirical values of $p$, $p_{n}$ and $p_{p}$ from population members in specific settings as has been done in western Kenya [39]. For the numerical examples, individual beliefs from low to high are used except in the case of a positive RDT result where the individual always has high trust in the test. Total subsidy cost (6) is influenced by the extent of the malaria problem among individuals visiting drug shops so the impact of different malaria prevalences on subsidy levels of RDT and ACT is also investigated.

To gain intuition on the subsidy sizes that are needed to fulfil the health planner’s objective for a range of different beliefs of the individual, a series of numerical examples or simulations are developed using the parameter values described above. The calculations are performed using linear programming methods to ensure that the costs of the health planner are minimized by finding the minimum subsidy levels of ACT and RDT that at the same time ensure that incentive constraints (1)–(5) listed in Additional file 3 hold for an individual with given beliefs and a given set of parameter values (from Table 1).^1^

## Results: optimal subsidies for RDT and ACT

**Fig. 2:**
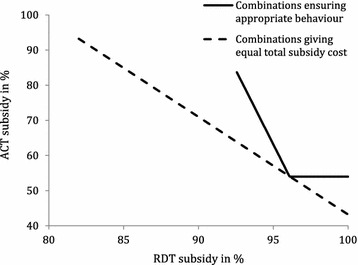
Optimal combination of RDT and ACT subsidies ensuring appropriate behaviour for a representative individual. Individual characterized by low belief that a fever is malaria ($p = 0.20$), low trust in negative RDT result ($p_{n} = 0.15$), high trust in positive RDT result ($p_{p} = 0.97$) and low malaria prevalence ($\bar{p} = 0.15$)

Figure 2 presents a situation where an individual has a low belief that the fever is malaria, a low trust in a negative RDT result, a high trust in a positive RDT result and with low parasite prevalence among individuals visiting drug shops. Such an individual may be incentivized always to purchase an RDT before treatment and buy an ACT medicine only in the case of a positive RDT result if the combined subsidies on RDT and ACT are on the solid line. For example, the individual will behave appropriately if the RDT subsidy is 93% and the ACT subsidy is 81% and also if the RDT subsidy is 97% and the ACT subsidy is 54%. Note that even if the RDT is free (100% subsidy) a positive ACT subsidy is required. In addition, the individual will only behave appropriately if the RDT subsidy is at least 93%; any RDT subsidy below this value will lead to inappropriate behaviour irrespective of the level of subsidy on the ACT—if the RDT is too expensive relative to ACT, the individual will go directly to buying ACT medicines without taking an RDT first.

The dotted line in Fig. 2 shows combinations of ACT and RDT subsidies giving equal total subsidy cost for the health planner (the sum of subsidy cost of ACT and RDT). The further to the south-west this line is situated, the lower the total subsidy cost. The optimal combination of subsidies from the health planner’s point of view is the point of tangency between the two lines at 96% subsidy on the RDT and 54% subsidy on the ACT since this will at the same time ensure appropriate behaviour of the individual and the lowest possible subsidy cost of the health planner. The total subsidy cost at this point is US$2.08 per individual.

Optimal subsidy combinations in situations of different beliefs of the individual and malaria prevalence are presented in Table 2. Among the beliefs investigated, it is not possible to ensure appropriate behaviour by subsidizing only ACT *or* RDT. The subsidy policy must be a combined subsidy on both commodities characterized by a high subsidy on the RDT of 80–96% of the retail price and a more moderate subsidy on the ACT in the range 54–76%. The intuition behind such a subsidy pattern is that in this model a low price of RDT is required to ensure that the individual is willing to purchase a test before treatment combined with a moderately reduced ACT price still high enough to ensure adherence to the RDT result. If the ACT price is too low, the individual may decide always to purchase an ACT medicine even if the RDT is negative and if the ACT price is too high, the individual may choose to purchase monotherapy even when the RDT is positive.

**Table 2:** Optimal combinations of RDT and ACT subsidies for different beliefs of a representative individual and malaria parasite prevalence

Individual beliefs with respect to:			Malaria prevalence ($$\bar{p}$$)	Subsidy in %		Total subsidy cost per individual in US$
Fever is malaria ($$p$$)	Negative RDT ($$p_{n}$$)	Positive RDT ($$p_{p}$$)		RDT	ACT	
0.20	0.03	0.97	0.15	88.4	54.0	1.94
0.20	0.03	0.97	0.35	88.4	54.0	2.28
0.20	0.03	0.97	0.50	88.4	54.0	2.54
0.20	0.03	0.97	0.70	88.4	54.0	2.88
0.20	0.15	0.97	0.15	96.1	54.0	2.08
0.20	0.15	0.97	0.35	96.1	54.0	2.42
0.20	0.15	0.97	0.50	96.1	54.0	2.68
0.20	0.15	0.97	0.70	96.1	54.0	3.02
0.40	0.10	0.97	0.15	80.0	76.0	1.93
0.40	0.10	0.97	0.35	94.5	54.0	2.39
0.40	0.10	0.97	0.50	94.5	54.0	2.65
0.40	0.10	0.97	0.70	94.5	54.0	2.99
0.40	0.35	0.97	0.15	–	–	–
0.40	0.35	0.97	0.35	–	–	–
0.40	0.35	0.97	0.50	–	–	–
0.40	0.35	0.97	0.70	–	–	–
0.60	0.15	0.97	0.15	82.9	68.3	1.93
0.60	0.15	0.97	0.35	82.9	68.3	2.36
0.60	0.15	0.97	0.50	82.9	68.3	2.69
0.60	0.15	0.97	0.70	98.1	54.0	3.05
0.60	0.55	0.97	0.15	–	–	–
0.60	0.55	0.97	0.35	–	–	–
0.60	0.55	0.97	0.50	–	–	–
0.60	0.55	0.97	0.70	–	–	–
0.80	0.20	0.97	0.15	90.1	60.6	2.01
0.80	0.20	0.97	0.35	90.1	60.6	2.40
0.80	0.20	0.97	0.50	90.1	60.6	2.68
0.80	0.20	0.97	0.70	90.1	60.6	3.06
0.80	0.75	0.97	0.15	–	–	–
0.80	0.75	0.97	0.35	–	–	–
0.80	0.75	0.97	0.50	–	–	–
0.80	0.75	0.97	0.70	–	–	–

– No solution

For some combinations of beliefs of the individual, there are no solutions to the problem meaning that no subsidies can be found to incentivize the individual to behave appropriately. This was found to be the case if the individual has a strong prior belief in being malaria positive (40% and above) and at the same time a weak belief in a negative test result.

The calculations performed further suggest that low confidence in a negative RDT result requires a higher RDT subsidy compared to high confidence while the ACT subsidy is not affected. No associations are apparent between RDT and ACT subsidy levels and the degree of belief that the fever is malaria and the malaria prevalence among individuals visiting drug shops. Finally, the total subsidy costs are higher for increasing malaria prevalence and for decreasing belief in a negative RDT result.

Sensitivity analyses are performed using the lower and upper bound parameter values in Table 1. Higher retail prices of ACT medicines and RDTs lead to higher subsidy costs (Table 3). However, a higher price on anti-malarial monotherapy may actually lead to lower required subsidies on RDT and ACT and lower subsidy cost, as a higher price on monotherapy makes it less attractive to follow strategies involving buying these drugs. Note that using the lowest bound estimate on ACT prices means that ACT should in fact be taxed and not subsidized to incentivize optimal behaviour.

**Table 3:** Sensitivity analysis of prices of ACT, RDT and monotherapy

	ACT price			RDT price			Monotherapy price		
	ACT %	RDT %	Cost	ACT %	RDT %	Cost	ACT %	RDT %	Cost
Best estimate	68.3	82.9	2.69	68.3	82.9	2.69	68.3	82.9	2.69
Lowest bound	−11.0	82.9	1.44	68.3	38.4	1.39	No solution	No solution	No solution
Highest bound	88.9	82.9	5.94	68.3	89.5	3.82	51.1	67.9	2.12

Results are presented for an individual with $p = 0.6$, $p_{n} = 0.15$, $p_{p} = 0.97$ and malaria prevalence is $\bar{p} = 0.5$

It is also investigated how changes in monotherapy effectiveness affect the results (Table 4). A lower effectiveness of monotherapy will, all else equal, make it less attractive for the individual to buy monotherapy and thus easier for the health planner to incentivize the use of ACT. However, the effect of monotherapy effectiveness on RDT uptake is not straightforward as a higher relative (perceived) effectiveness of ACT means that the individual needs a larger incentive to buy an RDT before buying an ACT medicine. The beliefs of the relative effectiveness of the different treatment types are, therefore, also important for reducing total subsidy costs.

**Table 4:** Sensitivity analysis of monotherapy effectiveness

Monotherapy effectiveness	ACT %	RDT %	Total subsidy cost per individual in US$
Best estimate (50%)	68.3	82.9	2.69
Lowest bound (20%)	52.9	69.4	2.17
Highest bound (70%)	No solution	No solution	No solution

All other parameters are best estimate parameter values from Table 1. Results are presented for an individual with $p = 0.6$, $p_{n} = 0.15$, $p_{p} = 0.97$ and malaria prevalence is $\bar{p} = 0.5$

## Discussion and conclusions

The simulations using the framework developed suggested that irrespective of the beliefs of the representative individual, the optimal subsidy policy of the health planner would involve a shared subsidy on RDT and ACT. In other words, the individual with fever would not be incentivized to behave appropriately through a subsidy on the RDT or the ACT alone. Even in a situation where the individual has high trust in both positive and negative RDT results, it would still be necessary to subsidize both the RDT and the ACT. Simulations further found that the optimal policy incorporated a high subsidy on RDT and a more moderate subsidy on ACT (Table 2). Previous empirical research has provided some support for a combined subsidy. Cohen et al. [21] provided subsidized RDTs to drug shops in Uganda but no subsidy on ACT treatment and found that among customers buying RDTs only 32% of RDT-positive patients purchased an ACT. Contrary to this, the introduction of both subsidized RDTs and ACT medicines in Ugandan drug shops resulted in high willingness to purchase an RDT before treatment and with almost all RDT-positive customers also buying an ACT and RDT-negative patients not buying an anti-malarial [40]. A similar study involving a combined subsidy among Kenyan drug shops also improved appropriate behaviour among drug shop customers but to a lesser extent [22]. These studies therefore point to different subsidy recommendations than the original AMFm approach which proposed subsidizing only ACT at a very high percentage of up to 95% of the manufacturer price [27]. The main objective of the latter was improving access to high quality ACT medicines and less concern for overprescription of ACT to patients with no malaria parasites in their blood [26].

It was found that a solution to the decision problem could not be identified in all situations including if the individual was highly convinced that his fever was malaria even before considering a test and at the same time had a very high distrust in a negative RDT result. Such an individual would prefer to purchase an anti-malarial without first taking a test as was also confirmed for some settings in a model-based study involving six African countries [41]. Qualitative research has confirmed that some patients and child caregivers indeed have confidence in their own ability to recognize malaria symptoms [37, 38]. In addition, perceived benefits of parasitological diagnosis among customers in the private sector are negatively affected when the risk of taking anti-malarials is perceived to be minimal, the concerns for delayed treatment of the true cause of fever if not malaria are minimal or believing more in an approach where different drugs are taken until one proves effective (diagnosis-by-treatment) [37, 38, 42]. If such perceptions are common, the subsidy instrument must be supplemented by a behaviour change communication campaign addressing unfortunate behaviours in a particular community.

A key parameter influencing the optimal subsidy structure in the present model is the degree of belief in a negative RDT result. The higher the mistrust in a negative RDT result, the higher a subsidy on RDTs is required. Mistrust in negative test results has been a matter of great concern for a long time in malaria care and several studies have indeed demonstrated a significant tendency to disregard negative RDT and microscopy results both among health providers and patients [22, 37, 43]. However, more recent studies indicate a higher belief in negative test results e.g. Mbonye et al. [40]: Following an information campaign on the advantages of RDTs and ACT treatment for malaria, RDTs were introduced in drug shops in an area of Uganda. The study found a high willingness to purchase a subsidized RDT among drug shop customers with fever and a nearly complete acceptance of negative RDT results as measured by the finding that almost all RDT-negative customers did not buy an ACT. This is encouraging since a high level of belief in the accuracy of the RDT will in the model require a lower RDT subsidy and lead to a lower overall subsidy cost.

The model developed for the presented analysis is a simplification in at least three respects. It was assumed that there is only one representative individual (or many identical individuals), that there are no drugs for non-malarial fevers offered at drug shops and that drug shops have a very simplified behaviour limited to wanting to sell RDTs and anti-malarials at the market price or at the subsidized price without any other considerations such as maximizing their own profit. One first possible expansion of the model could be allowing for many individuals with heterogeneous beliefs in for instance negative RDT results or the conviction that their fever is malaria. This would not change the health planner’s decision problem in principle, but instead of one set of constraints ensuring that the representative individual prefers the appropriate treatment strategy to any of the other strategies, it would require at set of constraints for each type of individual. It is also likely that the health planner is not able to find an RDT and ACT subsidy allocation that will simultaneously ensure appropriate behaviour in all drug shop customers. As shown above, individuals with certain beliefs cannot be incentivized into appropriate behaviour through the use of subsidies. The health planner will therefore have to decide on the minimum acceptable share of drug shop customers behaving appropriately.

Another possible extension to the model is assuming that a wider range of drugs relevant for fevers are available at drug shops such as antipyretics and antibiotics. Such an extension to the model would lead to an increase in the possible strategies of the individual due to a higher number of drugs and possibly also diagnostic tests. Identifying the optimal subsidy strategy is a significantly more complicated decision problem and will require further research.

A third possible extension to the model is allowing a more realistic behaviour of drug shops involving for instance consideration on how to maximize their profit. Drug shop behaviour may also be analysed under different market conditions facing drug shops in the community including monopoly, a situation with few competitors or many drug shops leading to perfect competition. Such extensions are likely to affect the assumption that the entire subsidy amount is passed on to customers. As a result, the health planner’s problem will be much more complicated to solve since it must be determined first what shares of the subsidies are passed on to the customers before the optimal combination of RDT and ACT subsidies can be identified.

## Additional files

**Additional file 1.** List of expected utility functions for all possible combinations of treatment after an individual has decided to purchase an RDT.

**Additional file 2.** Formal proof that six of the possible diagnosis-treatment strategies of an individual are dominated or suboptimal.

**Additional file 3.** Derivation of the conditions under which an individual will always choose to purchase an RDT followed by buying an ACT only if the test is positive.

## Footnotes

1 In practice, a linear optimization model was set up in Excel and the solver’s LP simplex function was applied.

## References

[1] WHO (2015). World malaria report 2015.

[2] White MT, Conteh L, Cibulskis R, Ghani AC (2011). Costs and cost-effectiveness of malaria control interventions—a systematic review. Malar J.

[3] Management Sciences for Health (2010). International drug price indicator guide 2009.

[4] WHO (2011). World malaria report 2011.

[5] O’Connell KA, Gatakaa H, Poyer S, Njogu J, Evance I, Munroe E (2011). Got ACTs? Availability, price, market share and provider knowledge of anti-malarial medicines in public and private sector outlets in six malaria-endemic countries. Malar J.

[6] Kangwana BB, Njogu J, Wasunna B, Kedenge SV, Memusi DN, Goodman CA (2009). Malaria drug shortages in Kenya: a major failure to provide access to effective treatment. Am J Trop Med Hyg.

[7] Zurovac D, Tibenderana JK, Nankabirwa J, Ssekitooleko J, Njogu JN, Rwakimari JB (2008). Malaria case-management under artemether–lumefantrine treatment policy in Uganda. Malar J..

[8] WHO (2012). World malaria report 2012.

[9] Goodman C, Brieger W, Unwin A, Mills A, Meek S, Greer G (2007). Medicine sellers and malaria treatment in sub-Saharan Africa: what do they do and how can their practice be improved?. Am J Trop Med Hyg.

[10] Patouillard E, Hanson KG, Goodman CA (2010). Retail sector distribution chains for malaria treatment in the developing world: a review of the literature. Malar J.

[11] Whitty CJM, Chandler C, Ansah E, Leslie T, Staedke SG (2008). Deployment of ACT antimalarials for treatment of malaria: challenges and opprtunities. Malar J.

[12] Wafula FN, Miriti EM, Goodman CA (2012). Examining characteristics, knowledge and regulatory practices of specialized drug shops in Sub-Saharan Africa: a systematic review of the literature. BMC Health Ser Res.

[13] Mbonye AK, Lal S, Cundill B, Hansen KS, Clarke S, Magnussen P (2013). Treatment of fevers prior to introducing rapid diagnostic tests for malaria in registered drug shops in Uganda. Malar J.

[14] Palafox B, Patouillard E, Tougher S, Goodman C, Hanson K, Kleinschmidt I (2016). Prices and mark-ups on antimalarials: evidence from nationally representative studies in six malaria-endemic countries. Health Policy Plan.

[15] Reyburn H, Mbatia R, Drakeley C, Carneiro I, Mwakasungula E, Mwerinde O (2004). Overdiagnosis of malaria in patients with severe febrile illness in Tanzania: a prospective study. BMJ.

[16] Perkins MD, Bell DR (2008). Working without a blindfold: the critical role of diagnostics in malaria control. Malar J.

[17] ACTwatch. Results and publications. Undated. http://www.actwatch.info/publications.

[18] Amexo M, Talhurst R, Barnish G, Bates I (2004). Malaria misdiagnosis: effects on the poor and vulnerable. Lancet.

[19] Mwanziva C, Shekalaghe S, Ndaro A, Mengerink B, Megiroo S, Mosha F (2008). Overuse of artemisinin-combination therapy in Mto wa Mbu (river of mosquitoes), an area misinterpreted as high endemic for malaria. Malar J.

[20] Ansah EK, Epokor M, Whitty CJM, Yeung S, Hansen KS (2014). Cost-effectiveness analysis of introducing RDTs for malaria diagnosis as compared to microscopy and presumptive diagnosis in central and peripheral public health facilities in Ghana. Am J Trop Med Hyg.

[21] Cohen J, Fink G, Berg K, Aber F, Jordan M, Maloney K (2012). Feasibility of distributing rapid diagnostic tests for malaria in the retail sector: evidence from an implementation study in Uganda. PLoS ONE.

[22] Cohen J, Dupas P, Schaner SG (2015). Price subsidies, diagnostic tests, and targeting of malaria treatment: evidence from a randomized controlled trial. Am Econ Rev.

[23] Kachur SP, Schulden J, Goodman CA, Kassala H, Elling BF, Khatib RA (2006). Prevalence of malaria parasitemia among clients seeking treatment for fever or malaria at drug stores in rural Tanzania 2004. Trop Med Int Health..

[24] Schellenberg D, Reyburn H, Yeung S, Bosman A, Snow S, Lansang MA, et al. Consultation on the economics and financing of universal access to parasitological confirmation of malaria. Geneva: The Global Fund; 2010. www.theglobalfund.org/documents/amfm/AMFm_EconFinancePreread_Appendix02_en.

[25] Briggs MA, Kalolella A, Bruxvoort K, Wiegand R, Lopez G, Festo C (2014). Prevalence of malaria parasitemia and purchase of artemisinin-based combination therapies (ACTs) among drug shop clients in two regions in Tanzania with ACT subsidies. PLoS ONE.

[26] Arrow K, Panosian C, Gelband H (2004). Saving lives, buying time: economics of malaria drugs in an age of resistance.

[27] Gelband H, Seiter A (2007). A global subsidy for antimalarial drugs. Am J Trop Med Hyg.

[28] Tougher S, Ye Y, Amuasi JH, Kourgueni IA, Thomson R, Goodman C (2012). Effect of the Affordable Medicines Facility—malaria (AMFm) on the availability, price, and market share of quality-assured artemisinin-based combination therapies in seven countries: a before-and-after analysis of outlet survey data. Lancet.

[29] Sabot OJ, Mwita A, Cohen JM, Ipuge Y, Gordon M, Bishop D (2009). Piloting the global subsidy: the impact of subsidized artemisinin-based combination therapies distributed through private drug shops in rural Tanzania. PLoS ONE.

[30] Kangwana BP, Kedenge S, Noor AM, Alegana VA, Nyandigisi AJ, Pandit J (2011). The impact of retail-sector delivery of artemether–lumefantrine on malaria treatment of children under five in Kenya: a cluster randomized controlled trial. PLoS Med.

[31] Fink G, Dickens WT, Jordan M, Cohen JL (2014). Access to subsidized ACT and malaria treatment—evidence from the first year of the AMFm program in six districts in Uganda. Health Policy Plan.

[32] WHO (2010). Guidelines for the treatment of malaria.

[33] de Oliveira AM, Skarbinski J, Ouma PO (2009). Performance of malaria rapid diagnostic tests as part of routine malaria case management in Kenya. Am J Trop Med Hyg.

[34] Baiden F, Webster J, Tivura M, Delimini R, Berko Y, Amenga-Etego S (2012). Accuracy of rapid tests for malaria and treatment outcomes for malaria and non-malaria cases among under-five children in rural Ghana. PLoS ONE.

[35] The Global Fund. Board approves integration of AMFm into core global fund grant processes. 2012. http://www.theglobalfund.org/en/mediacenter/newsreleases/2012-11-15_Board_Approves_Integration_of_AMFm_into_Core_Global_Fund_Grant_Processes.

[36] Hansen KS, Pedrazzoli D, Mbonye A, Clarke S, Cundill B, Magnussen P (2013). Willingness-to-pay for a rapid malaria diagnostic test and artemisinin-based combination therapy from private drug shops in Mukono district, Uganda. Health Policy Plan..

[37] Chandler CIR, Hall-Clifford R, Asaph T, Magnussen P, Clarke S, Mbonye AK (2011). Introducing malaria rapid diagnostic tests at registered drug shops in Uganda: limitations of diagnostic testing in the reality of diagnosis. Soc Sci Med.

[38] Cohen J, Cox A, Dickens W, Maloney K, Lam F, Fink G (2015). Determinants of malaria diagnostic uptake in the retail sector: qualitative analysis from focus groups in Uganda. Malar J.

[39] Prudhomme O’Meara W, Laktabai J, Mohanan M, Turner E, Maffioli E, Platt A, et al. Targeting antimalarial subsidies to confirmed cases in the retail sector—testing a diagnosis-dependent voucher scheme in western Kenya. Annual Meeting of the American Society of Tropical Medicine and Hygiene 2015 (Abstract 1187); 2015.

[40] Mbonye A, Magnussen P, Lal S, Hansen K, Cundill B, Chandler C (2015). Cluster randomised trial introducing rapid diagnostic tests into the private health sector in Uganda: the impact on appropriate targeting of malaria treatment. PLoS ONE.

[41] Basu S, Modrek S, Bendavid E (2014). Comparing decisions for malaria testing and presumptive treatment: a net health benefit analysis. Med Decis Mak.

[42] Mbonye AK, Ndyomugyenyi R, Turinde A, Magnussen P, Clarke S, Chandler C (2010). The feasibility of introducing rapid diagnostic tests for malaria in drug shops in Uganda. Malar J.

[43] Hamer D, Ndhlovu M, Zurovac D, Fox M (2007). Improved diagnostic testing and malaria treatment practices in Zambia. JAMA.

[44] Goodman C, Kachur SP, Abdulla S, Bloland P, Mills A (2009). Concentration and drug prices in the retail market for malaria treatment in rural Tanzania. Health Econ.

[45] Morel CM, Lauer JA, Evans DB (2005). Cost-effectiveness analysis of strategies to combat malaria in developing countries. BMJ.

[46] Mueller O, Razum O, Traore C, Kouyate B (2004). Community effectiveness of chloroquine and traditional remedies in the treatment of young children with falciparum malaria in rural Burkina Faso. Malar J.

[47] Sinclair D, Zani B, Donegan S, Olliaro P, Garner P (2009). Artemisinin-based combination therapy for treating uncomplicated malaria. Cochrane Database Syst Rev.

[48] Björkman A, Mårtensson A (2010). Risks and benefits of targeted malaria treatment based on rapid diagnostic test results. Clin Infect Dis.

[49] Bisoffi Z, Sirima SB, Menten J, Pattaro C, Angheben A, Gobbi F (2010). Accuracy of a rapid diagnostic test on the diagnosis of malaria infection and of malaria-attributable fever during low and high transmission season in Burkina Faso. Malar J.

[50] Medicines for Malaria Venture (2008). Understanding the antimalarials market: Uganda 007—an overview of the supply side.

