# Supplementary material for: Optimal price subsidies for appropriate malaria testing and treatment behaviour
Source: Malar J. 2016 Nov 4;15:534. doi: 10.1186/s12936-016-1582-1 (PMC5097430; doi:10.1186/s12936-016-1582-1)
Supplement: Supplementary file 1 — Additional file 1. List of expected utility functions for all possible combinations of treatment after an individual has decided to purchase an RDT. [file 12936_2016_1582_MOESM1_ESM.docx]

**Additional file 1:** List of expected utility functions for all possible combinations of treatment after an individual has decided to purchase an RDT.

Without loss of generality and to simplify the notation in subsequent equations we normalize the utility of the two health states, “malaria” and “not malaria” such that $\tilde{V}_{m}=0$ and $\tilde{V}_{nm}=1,$ where the tilde indicates that the values are normalized. To convert the monetary prices ($C_{MT}$, $C_{ACT}$ and $C_{RDT}$) into a price measure comparable to the normalized utility model we use a linear transformation where we divide the monetary (US$) price with the individual’s willingness to pay (WTP) for avoiding malaria illness to create a normalized price of anti-malarials and RDT:

$\tilde{C}=\frac{C}{V_{nm}-V_{m}}$.

The expected utility of a strategy of buying first an RDT followed by the purchase of a course of ACT if the test is positive and not purchase any drugs if the test is negative is presented in the main text. As indicated by the decision tree in Figure 1, the individual may however choose other combinations of treatment after the test like for instance buying an anti-malarial monotherapy if the test is positive and not purchase any drug if the test is positive. The expected utility of all possible combinations of treatment after the RDT has been listed below (again, the tilde indicates that the values are normalized).

A1) Buying an ACT if the test is positive and not purchase any drugs if the test is negative (normalized version of the equation presented in the main text):

$\tilde{U}\left( S_{(ACT,NO)}^{RDT} \right)=p^{*}[p_{p}E_{ACT}+\left( 1-p_{p} \right)-\tilde{C}_{ACT}]+\left( 1-p^{*} \right)\left( 1-p_{n} \right)-\tilde{C}_{RDT}$.

A2) Buying an anti-malarial monotherapy if the test is positive and also buying monotherapy if the test is negative:

$\tilde{U}\left( S_{\left( MT,MT \right)}^{RDT} \right)=p^{*}\left[ p_{p}E_{MT}+\left( 1-p_{p} \right)-\tilde{C}_{MT} \right]+\left( 1-p^{*} \right)\left[ p_{n}E_{MT}+\left( 1-p_{n} \right)-\tilde{C}_{MT} \right]-\tilde{C}_{RDT}$.

A3) Buying an anti-malarial monotherapy if the test is positive and buying an ACT if the test is negative:

$\tilde{U}\left( S_{\left( MT,ACT \right)}^{RDT} \right)=p^{*}\left[ p_{p}E_{MT}+\left( 1-p_{p} \right)-\tilde{C}_{MT} \right]+\left( 1-p^{*} \right)\left[ p_{n}E_{ACT}+\left( 1-p_{n} \right)-\tilde{C}_{ACT} \right]-\tilde{C}_{RDT}$.

A4) Buying an anti-malarial monotherapy if the test is positive and not purchasing any drugs if the test is negative:

$\tilde{U}\left( S_{(MT,NO)}^{RDT} \right)=p^{*}[p_{p}E_{MT}+\left( 1-p_{p} \right)-\tilde{C}_{MT}]+\left( 1-p^{*} \right)\left( 1-p_{n} \right)-\tilde{C}_{RDT}$.

A5) Buying an ACT if the test is positive and buying an anti-malarial monotherapy if the test is negative:

$\tilde{U}\left( S_{(ACT,MT)}^{RDT} \right)=p^{*}\left[ p_{p}E_{ACT}+\left( 1-p_{p} \right)-\tilde{C}_{ACT} \right]+\left( 1-p^{*} \right)\left[ p_{n}E_{MT}+\left( 1-p_{n} \right)-\tilde{C}_{MT} \right]-\tilde{C}_{RDT}$.

A6) Buying an ACT if the test is positive and also buying an ACT if the test is negative:

$\tilde{U}\left( S_{(ACT,ACT)}^{RDT} \right)= p^{*}\left[ p_{p}E_{ACT}+\left( 1-p_{p} \right)-\tilde{C}_{ACT} \right]+\left( 1-p^{*} \right)[p_{n}E_{ACT}+\left( 1-p_{n} \right)-\tilde{C}_{ACT}]-\tilde{C}_{RDT}$.

A7) Buying no drugs if the test is positive and buying an anti-malarial monotherapy if the test is negative:

$\tilde{U}\left( S_{(NO,MT)}^{RDT} \right)=p^{*}\left( 1-p_{p} \right)+\left( 1-p^{*} \right)\left[ p_{n}E_{MT}+\left( 1-p_{n} \right)-\tilde{C}_{MT} \right]-\tilde{C}_{RDT}$.

A8) Buying no drugs if the test is positive and buying an ACT if the test is negative:

$\tilde{U}\left( S_{(NO,ACT)}^{RDT} \right)=p^{*}(1-p_{p})+\left( 1-p^{*} \right)[p_{n}E_{ACT}+\left( 1-p_{n} \right)-\tilde{C}_{ACT}]-\tilde{C}_{RDT}$.

A9) Buying no drugs if the test is positive and also buying no drugs if the test is negative:

$\tilde{U}\left( S_{(NO,NO)}^{RDT} \right)=p^{*}(1-p_{p})+\left( 1-p^{*} \right)\left( 1-p_{n} \right)-\tilde{C}_{RDT}$.
