# Supplementary material for: Optimal price subsidies for appropriate malaria testing and treatment behaviour
Source: Malar J. 2016 Nov 4;15:534. doi: 10.1186/s12936-016-1582-1 (PMC5097430; doi:10.1186/s12936-016-1582-1)
Supplement: Supplementary file 2 — Additional file 2. Formal proof that six of the possible diagnosis-treatment strategies of an individual are dominated or suboptimal. [file 12936_2016_1582_MOESM2_ESM.docx]

**Additional file 2:** Formal proof that six of the possible diagnosis-treatment strategies of an individual are dominated or suboptimal.

This additional file proofs that six of the strategies involving purchasing an RDT are dominated or suboptimal, and thus can be eliminated by a rational decision maker. The strategies are: (1) $S_{(ACT,ACT)}^{RDT}$, (2) $S_{(MT,MT)}^{RDT}$, (3) $S_{(NO,NO)}^{RDT}$, (4) $S_{(MT,ACT)}^{RDT}$, (5) $S_{(NO,ACT)}^{RDT}$ and (6) $S_{(NO,MT)}^{RDT}$.

In contrast to strategies (1)-(3), each strategy (4)-(6) is not dominated by another strategy regardless of parameters. However the strategies (4)-(6) are still suboptimal in the sense that each of them is always inferior to another strategy (but which is better depends on the parameters).

Ad. (1)-(3):

$S_{(ACT,ACT)}^{RDT}$ is dominated by $S_{ACT}$ :

$$\tilde{U}\left( S_{ACT} \right)>\tilde{U}(S_{(ACT,ACT)}^{RDT})\overset{\Leftrightarrow}{}$$

$${{pE}_{ACT}+ \left( 1-p \right)- \tilde{C}_{ACT}>p}^{*}\left\{ p_{p}E_{ACT}+ \left( 1-p_{p} \right)- \tilde{C}_{ACT} \right\}+ \left( 1-p^{*} \right)\left\{ p_{n}E_{ACT}+ \left( 1-p_{n} \right)- \tilde{C}_{ACT} \right\}- \tilde{C}_{RDT} \overset{\Leftrightarrow}{}$$

$$\tilde{C}_{RDT}>0.$$

In a similar way, it can be shown that $S_{(MT,MT)}^{RDT}$ is dominated by $S_{MT}$, and $S_{(NO,NO)}^{RDT}$ is dominated by $S_{NO}$.

Ad (4):

For any combination of parameter values, $S_{(MT,ACT)}^{RDT}$ has lower expected utility than either $S_{ACT}$ or $S_{MT}$:

If $S_{(MT,ACT)}^{RDT}$ has lower expected utility than $S_{ACT}$ then:

$$\tilde{U}\left( S_{ACT} \right)>\tilde{U}(S_{(MT,ACT)}^{RDT})\overset{\Leftrightarrow}{}$$

$${{pE}_{ACT}+\left( 1-p \right)-\tilde{C}_{ACT}>p}^{*}\left[ p_{p}E_{MT}+\left( 1-p_{p} \right)-\tilde{C}_{MT} \right]+$$

$$\left( 1-p^{*} \right)[p_{n}E_{ACT}+\left( 1-p_{n} \right)-\tilde{C}_{ACT}]-\tilde{C}_{RDT}\overset{\Leftrightarrow}{}$$

$\tilde{C}_{RDT}>p^{*}{[\left( \tilde{C}_{ACT}-\tilde{C}_{MT} \right)-p}_{p}\left( E_{ACT}-E_{MT} \right)]$.

If $S_{(MT,ACT)}^{RDT}$ has lower expected utility than $S_{MT}$ then:

$$\tilde{U}\left( S_{MT} \right)>\tilde{U}(S_{(MT,ACT)}^{RDT})\overset{\Leftrightarrow}{}$$

$${{pE}_{MT}+\left( 1-p \right)-\tilde{C}_{MT}\geq p}^{*}\left[ p_{p}E_{MT}+\left( 1-p_{p} \right)-\tilde{C}_{MT} \right]$$

$$+\left( 1-p^{*} \right)[p_{n}E_{ACT}+\left( 1-p_{n} \right)-\tilde{C}_{ACT}]-\tilde{C}_{RDT}\overset{\Leftrightarrow}{}$$

$\tilde{C}_{RDT}>-\left( 1-p^{*} \right)[{\left( \tilde{C}_{ACT}-\tilde{C}_{MT} \right)-p}_{n}\left( E_{ACT}-E_{MT} \right)]$.

We note from above that if $S_{ACT}$ has lower expected utility than $S_{(MT,ACT)}^{RDT}$ then we have $\left( \tilde{C}_{ACT}-\tilde{C}_{MT} \right)>p_{p}\left( E_{ACT}-E_{MT} \right).$ Since $p_{n}<p_{p}$ this implies that $\left( \tilde{C}_{ACT}-\tilde{C}_{MT} \right)>p_{n}\left( E_{ACT}-E_{MT} \right).$ This means that $-\left( 1-p^{*} \right)\left[ \left( \tilde{C}_{ACT}-\tilde{C}_{MT} \right)-p_{n}\left( E_{ACT}-E_{MT} \right) \right]<0$ implying instead that $S_{MT}$ has higher expected utility than $S_{RDT(MT,ACT)}$. Therefore, if $S_{(MT,ACT)}^{RDT}$ has lower expected utility than $S_{ACT}$ then instead $S_{MT}$ will have higher expected utility than $S_{(MT,ACT)}^{RDT}$.

Ad (5):

For any combination of parameter values, $S_{(NO,ACT)}^{RDT}$ has lower expected utility than either $S_{ACT}$ or $S_{NO}$:

If $S_{(NO,ACT)}^{RDT}$ has lower expected utility than $S_{ACT}$ then:

$$\tilde{U}\left( S_{ACT} \right)>\tilde{U}(S_{(NO,ACT)}^{RDT})\overset{\Leftrightarrow}{}$$

$${{pE}_{ACT}+ \left( 1-p \right)- \tilde{C}_{ACT}>p}^{*}\left( 1-p_{p} \right)+\left( 1-p^{*} \right)[p_{n}E_{ACT}+\left( 1-p_{n} \right)-\tilde{C}_{ACT}]-\tilde{C}_{RDT}\overset{\Leftrightarrow}{}$$

$\tilde{C}_{RDT}>p^{*}{(\tilde{C}}_{ACT}-p_{p}E_{ACT})$.

If $S_{(NO,ACT)}^{RDT}$ has lower expected utility than $S_{NO}$ then:

$$\tilde{U}\left( S_{NO} \right)>\tilde{U}(S_{(NO,ACT)}^{RDT})\overset{\Leftrightarrow}{}$$

$$(1-p)\geq p^{*}\left( 1-p_{p} \right)+\left( 1-p^{*} \right)[p_{n}E_{ACT}+\left( 1-p_{n} \right)-\tilde{C}_{ACT}] - \tilde{C}_{RDT}\overset{\Leftrightarrow}{}$$

$\tilde{C}_{RDT}>-\left( 1-p^{*} \right)[\tilde{C}_{ACT}-p_{n}E_{ACT}]$.

The strategy $S_{(NO,ACT)}^{RDT}$ can be eliminated since either $S_{ACT}$ or $S_{NO}$ is always better. To see this, note that if $S_{(NO,ACT)}^{RDT}$ has not lower expected utility than $S_{ACT}$, then ${(\tilde{C}}_{ACT}-p_{p}E_{ACT})>0.$ But since $p_{n}<p_{p}$ we have ${(\tilde{C}}_{ACT}-p_{n}E_{ACT})>0$and also that $-\left( 1-p^{*} \right)[\tilde{C}_{ACT}-p_{n}E_{ACT}]<0$ implying that $S_{NO}$ has higher expected utility than $S_{(NO,ACT)}^{RDT}$. Therefore, if $S_{(NO,ACT)}^{RDT}$ does not have lower expected utility than $S_{ACT}$ then instead $S_{NO}$ will have higher expected utility than $S_{(NO,ACT)}^{RDT}$.

Ad (6):

For any combination of parameter values, $S_{(NO,MT)}^{RDT}$ has lower expected utility than either $S_{MT}$ or $S_{NO}$:

If $S_{(NO,MT)}^{RDT}$ has lower expected utility than $S_{MT}$ then:

$$\tilde{U}\left( S_{MT} \right)>\tilde{U}(S_{(NO,MT)}^{RDT})\overset{\Leftrightarrow}{}$$

$$pE_{MT}+\left( 1-p \right)-\tilde{C}_{MT}>p^{*}\left( 1-p_{p} \right)+\left( 1-p^{*} \right)[p_{n}E_{MT}+\left( 1-p_{n} \right)-\tilde{C}_{MT}] - \tilde{C}_{RDT}\overset{\Leftrightarrow}{}$$

$\tilde{C}_{RDT}>p^{*}\left( \tilde{C}_{MT}-p_{p}E_{MT} \right)$.

If $S_{(NO,MT)}^{RDT}$ has lower expected utility than $S_{NO}$ then:

$$\tilde{U}\left( S_{NO} \right)>\tilde{U}(S_{(NO,MT)}^{RDT}S_{RDT(NO,MT)})\overset{\Leftrightarrow}{}$$

$$\left( 1-p \right)>p^{*}\left( 1-p_{p} \right)+\left( 1-p^{*} \right)[p_{n}E_{MT}+\left( 1-p_{n} \right)-\tilde{C}_{MT}] - \tilde{C}_{RDT}\overset{\Leftrightarrow}{}$$

$\tilde{C}_{RDT}>-\left( 1-p^{*} \right)[\tilde{C}_{MT}-p_{n}E_{MT}]$.

Strategy $S_{(NO,MT)}^{RDT}$ can be eliminated, since we always have that *either* $S_{MT}$ or $S_{NO}$ is better. If $S_{(NO,MT)}^{RDT}$ does not have lower expected utility than $S_{MT}$, we have that ${(\tilde{C}}_{MT}-p_{p}E_{MT})>0$. But since $p_{n}<p_{p}$ we then also have ${(\tilde{C}}_{MT}-p_{n}E_{MT})>0$ and further that $-\left( 1-p^{*} \right)[\tilde{C}_{MT}-p_{n}E_{MT}]<0$ implying that $S_{NO}$ has higher expected utility than $S_{(NO,MT)}^{RDT}$.
