# Supplementary material for: Optimal price subsidies for appropriate malaria testing and treatment behaviour
Source: Malar J. 2016 Nov 4;15:534. doi: 10.1186/s12936-016-1582-1 (PMC5097430; doi:10.1186/s12936-016-1582-1)
Supplement: Supplementary file 3 — Additional file 3. Derivation of the conditions under which an individual will always choose to purchase an RDT followed by buying an ACT only if the test is positive. [file 12936_2016_1582_MOESM3_ESM.docx]

**Additional file 3:** Derivation of the conditions under which an individual will always choose to purchase an RDT followed by buying an ACT only if the test is positive.

The main text discussed the condition under which the individual has higher expected utility from following strategy $S_{(ACT,NO)}^{RDT}$ rather than strategy $S_{ACT}$ leading the individual to always prefer the former strategy of the two. Using normalized utilities, the condition can be written as:

$$\tilde{U}\left( S_{(ACT,NO)}^{RDT} \right)>\tilde{U}\left( S_{ACT} \right)\overset{\Leftrightarrow}{}$$

$$p^{*}\left[ p_{p}E_{ACT}+\left( 1-p_{p} \right)-\tilde{C}_{ACT} \right]+\left( 1-p^{*} \right)\left( 1-p_{n} \right)-\tilde{C}_{RDT}>pE_{ACT}+\left( 1-p \right)-\tilde{C}_{ACT} \overset{\Leftrightarrow}{}$$

(1) $\left( 1-p^{*} \right)\tilde{C}_{ACT}-\tilde{C}_{RDT}>(1-p^{*})p_{n}E_{ACT}$

where the prices include the subsidies. For example $\tilde{C}_{ACT}=\frac{C_{ACT}-\beta^{ACT}}{V_{NM}-V_{M}}$.

Conditions required for ensuring that the individual prefers strategy $S_{(ACT,NO)}^{RDT}$ to all other possible strategies may be derived as follows:

Strategy $S_{(ACT,NO)}^{RDT}$ versus strategy $S_{MT}$:

$$\tilde{U}\left( S_{(ACT,NO)}^{RDT} \right)>\tilde{U}(S_{MT})\overset{\Leftrightarrow}{}$$

$$p^{*}\left[ p_{p}E_{ACT}+\left( 1-p_{p} \right)-\tilde{C}_{ACT} \right]+\left( 1-p^{*} \right)\left( 1-p_{n} \right)-\tilde{C}_{RDT}>pE_{MT}+\left( 1-p \right)-\tilde{C}_{MT}\overset{\Leftrightarrow}{}$$

(2) $p^{*}p_{p}E_{ACT}-\left[ p^{*}p_{p}+\left( 1-p^{*} \right)p_{n} \right]E_{MT}>\tilde{C}_{RDT}+p^{*}\tilde{C}_{ACT}-\tilde{C}_{MT}$.

Strategy $S_{(ACT,NO)}^{RDT}$ versus strategy $S_{NO}$:

$$\tilde{U}\left( S_{(ACT,NO)}^{RDT} \right)>\tilde{U}(S_{NO})\overset{\Leftrightarrow}{}$$

$$p^{*}\left[ p_{p}E_{ACT}+\left( 1-p_{p} \right)-\tilde{C}_{ACT} \right]+\left( 1-p^{*} \right)\left( 1-p_{n} \right)-\tilde{C}_{RDT}>\left( 1-p \right)\overset{\Leftrightarrow}{}$$

(3) $p^{*}p_{p}E_{ACT}>\tilde{C}_{RDT}+p^{*}\tilde{C}_{ACT}$.

Strategy $S_{(ACT,NO)}^{RDT}$ versus strategy $S_{(ACT,MT)}^{RDT}$:

$$\tilde{U}\left( S_{(ACT,NO)}^{RDT} \right)>\tilde{U}(S_{(ACT,MT)}^{RDT})\overset{\Leftrightarrow}{}$$

$$p^{*}\left[ p_{p}E_{ACT}+\left( 1-p_{p} \right)-\tilde{C}_{ACT} \right]+\left( 1-p^{*} \right)\left( 1-p_{n} \right)-\tilde{C}_{RDT}>p^{*}[p_{p}E_{ACT}+\left( 1-p_{p} \right)-\tilde{C}_{ACT}]+\left( 1-p^{*} \right)[p_{n}E_{MT}+\left( 1-p_{n} \right)-\tilde{C}_{MT}]-\tilde{C}_{RDT}\overset{\Leftrightarrow}{}$$

(4) $\tilde{C}_{MT}>p_{n}E_{MT}$.

Strategy $S_{(ACT,NO)}^{RDT}$ versus $S_{(MT,NO)}^{RDT}$:

$$\tilde{U}\left( S_{(ACT,NO)}^{RDT} \right)>\tilde{U}(S_{(MT,NO)}^{RDT})\overset{\Leftrightarrow}{}$$

$$p^{*}\left[ p_{p}E_{ACT}+\left( 1-p_{p} \right)-\tilde{C}_{ACT} \right]+\left( 1-p^{*} \right)\left( 1-p_{n} \right)-\tilde{C}_{RDT}>p^{*}[p_{p}E_{MT}+\left( 1-p_{p} \right)-\tilde{C}_{MT}]+\left( 1-p^{*} \right)\left( 1-p_{n} \right)-\tilde{C}_{RDT}\overset{\Leftrightarrow}{}$$

(5) $p_{p}\left( E_{ACT}-E_{MT} \right)>\left( \tilde{C}_{ACT}-\tilde{C}_{MT} \right)$.
